# An Ethnological Phenomenon

**Published:** 1880-08

**Authors:** Harvey L. Byrd

**Affiliations:** Formerly Professor of Materia Medica and Therapeutics, and Principles and Practice of Medicine; and late Professor of Obstetrics, and of the Disease of Women and Children., etc., etc., Baltimore, Md.


ARTICLE II.

### AN ETHNOLOGICAL PHENOMENON.

BY HARVEY L. BYRD A. M., M. D.

Formerly Professor of Materia Medica and Therapeutics, and Principles and Practice of Medicine; and late Professor of Obstetrics, and of the Disease of Women and Children., etc , etc., Baltimore, Md.

So constant and unvarying have been the appearance of a dark or blackened condition of the genitalia, in infants of mixed caucasian and negro blood, in an experience of more than thirty years, that I am

*Packard's edition of Malgaigne, vol. 1, p. 324.

†F. H. Hamilton: Fractures and Dislocation, second edition, p. 129.

led to believe it of universal occurrence; so long, at least, as a trace of negro blood continues to be mingled with that of the white progenitor, in its future epigone.

In the hybrid, mulatto, the scrotum of the male will be found dark, or black, and the vulva of the female child will be of the same color. Thus, in the offspring of a white male and negro female, the scrotum will be found as dark as the mother, whilst the remainder of the body will be of the mulatto hue, or a commingling of the white and black color of its parents; and where the sex of the offspring of such parents is female, the dark color of the mother will be found to cover the vulva, and the usual mulatto complexion, all other portions of its person. This darkness of the genitalia, is found also in the offspring of the mulatto woman and white man, and in the children of a quadroon by a caucasian father, and even in those of the octoroon, sired or begotten by a white man. How much farther it is transmitted I have had no opportunity of knowing; but thus far it is well defined, and unmistakable, in the two latter removes, even where the skin and all the usual characteristics of the highest blonde type, are prominent and distinguishable. After discovering it for the first time in an infant mulatto, my observations were continued with all the care attainable, under various circumstances; in the newly born, the young, the middle aged, and in the cases each, of an aged mulatto man and woman. The discoleration seems, therefore, to be prominent, as well as constant, in the mixed blooded descendants of the white and black races. Whether the offspring of caucasian women and negro men, present the same peculiarity, I have had no means of ascertaining; as hapily for our race, such cases of miscegenation and degradation, of caucasian (white) women in this country, are of exceedingly rare occurrence. Inquiry among intelligent negro mid-wives, in some of our southern cities, confirms my own observation of this phenomenon in every particular. How much a knowledge of this ethnological fact, for fact it is, may be of value in medico-legal science and future race investigation, can only be left to conjecture; but that it will be found, a factor of value in each, can hardly admit of reasonable doubt even now.

